# “Seat of the soul”? The structure and function of the pineal gland in women with alleged spirit possession—Results of two experimental studies

**DOI:** 10.1002/brb3.1693

**Published:** 2020-06-07

**Authors:** Marco Aurélio Vinhosa Bastos, Paulo Roberto Haidamus de Oliveira Bastos, Loyná Euá Flores e Paez, Edna Oliveira de Souza, Danielle Bogo, Renata Trentin Perdomo, Renata Boschi Portella, Jorge Guilherme Okanobo Ozaki, Décio Iandoli, Giancarlo Lucchetti

**Affiliations:** ^1^ Postgraduate Program in Health and Development Federal University of Mato Grosso do Sul Campo Grande Brazil; ^2^ School of Medicine Federal University of Mato Grosso do Sul Campo Grande Brazil; ^3^ ABRAPE Campo Grande Brazil; ^4^ School of Pharmaceutical Sciences Federal University of Mato Grosso do Sul Campo Grande Brazil; ^5^ Sonimed Imaging Clinic Campo Grande Brazil; ^6^ School of Medicine Anhanguera‐Uniderp University Campo Grande Brazil; ^7^ School of Medicine Federal University of Juiz de Fora Juiz de Fora Brazil

**Keywords:** 6‐sulfatoxymelatonin, dissociation, mediumship, pineal gland, pituitary gland, stress reactivity

## Abstract

**Background:**

Cultural traditions attribute to pineal gland an important role for spiritual experiences. Mediumship and spirit possession are cultural phenomena found worldwide which have been described as having dissociative and psychotic‐like characteristics, but with nonpathological aspects. A sympathetic activation pattern in response to spirit possession has been reported in some studies, but empirical data on pineal gland is scarce in this context.

**Methods:**

We aimed to investigate pineal gland and pituitary volumes, as well as urinary 6‐sulfatoxymelatonin levels in 16 alleged mediums (Medium Group‐MG) compared with 16 healthy nonmedium controls (Control Group) (Experiment 1). Furthermore, we aimed to evaluate urinary 6‐sulfatoxymelatonin and stress reactivity in GM (*n* = 10) under different physiological conditions (Experiment 2).

**Results:**

In Experiment 1, MG presented higher scores of anomalous experiences, but there were no between‐group differences regarding mental health or subjective sleep quality. Similar pineal gland and pituitary volumes were observed between groups. There were no between‐group differences in urinary 6‐sulfatoxymelatonin collected under equivalent baseline conditions. In Experiment 2, the rise of anxiety and heart rate in response to mediumistic experience was intermediate between a nonstressful control task (reading) and a stressful control task (Trier Social Stress Test—TSST). No significant differences were observed in 6‐sulfatoxymelatonin urinary levels between the three conditions. The pattern of stress reactivity during the TSST was normal, but with an attenuated salivary cortisol response.

**Conclusion:**

The normal neuroimaging and stress reactivity findings in MG contrast with the abnormal results usually observed in subjects with psychotic and dissociative disorders.

## Graphical abstract

Pineal and pituitary volumes similar in alleged mediums and nonmedium controls. Baseline 6‐sulfatoxymelatonin similar in alleged mediums and nonmedium controls. Subjective sleep quality similar in alleged mediums and nonmedium controls. Normal pattern of laboratory stress reactivity in alleged mediums. Results contrast to reports for with psychotic and dissociative disorders.

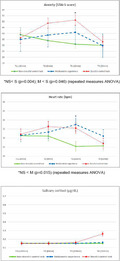

## 1 INTRODUCTION

The pineal gland (PG) is a small solid organ with an interhemispheric location at the base of the brain, in the midline roof of the third ventricle. PG's main byproduct is melatonin (MLT), a hormone derived from serotonin with an important regulating role in the sleep‐wake cycle, also exerting antioxidant and neuroprotective actions (Hardeland et al., 2011; Stehle et al., 2011). Most of the advances on the knowledge regarding PG's function is recent, from the twentieth century forth, but this gland has long been associated with spiritual issues (Lokhorst, 2018).

The first detailed descriptions of the PG and the first speculations as to its functions are found in the writings of Galen (AD 130–210). Galen believed in the “pneumatic‐ventricular” theory, which proposed that imagination, reasoning, memory, and body movements themselves were due to the circulation of volatile vaporous substances (“psychic pneuma”) inside the cerebral ventricular system (Lokhorst, 2018). Influenced by these theories and considering the anatomical location of the gland, Galen stated that the PG could be one of the brain structures to play a valvular role, allowing or preventing the passage of subtle fluids within the ventricular system (Lokhorst, 2018; Lucchetti, Daher, Iandoli, Gonçalves, & Lucchetti, 2013). It was only in the XVI century that the Italian anatomist Niccolò Massa (1485–1569) discovered and described that the cerebral ventricles were not filled with spiritual vapors, but with liquid (cerebrospinal fluid). Since then, all theories proposing the flow of spiritual fluids within the cerebral ventricles, as well as any function of PG in regulating this flow, have been rejected (Lokhorst, 2018).

However, the French philosopher and mathematician René Descartes (1596–1650), who also held a strong interest in anatomy and physiology, and who made important contributions regarding “philosophy of mind” and “mind–body theories,” kept on supporting the pneumatic‐ventricular model. Also, he tried to explain most of our mental life in terms of processes involving the PG, stating that is was the “seat of the soul” (Lokhorst, 2018; López‐Muñoz, Molina, Rubio, & Alamo, 2011). In the same vein, Hindu literature, yoga (Rama, Ballentine, & Ajaya, 2007), Spiritism religion (Lucchetti et al., 2013) and the esoteric literature (Luke, 2011; Strassman, 2001) bring speculations that the PG would have an important role to transcendence and contact with “higher energies.” Unconventional theories stemming from sociocultural traditions can generate hypotheses to be tested in scientific studies (Lucchetti et al., 2013).

Mediumship can be defined as the alleged ability to communicate regularly with deceased personalities (Rock, 2013). This is a phenomenon found in many societies around the world (Hunter & Luke, 2014), and it can manifest itself in many ways, such as listening or seeing spirits, speaking, or writing under the influence of spirits, spirit possession, among others. Mediumistic experiences have been described as having dissociative and psychotic‐like characteristics, but with nonpathological aspects, not being considered psychiatric disorders (Mainieri et al., 2017). As these occurrences are perceived as sensory but lack a material source, some clinicians tend to define them as hallucinations (Luhrmann, 2011) but, as the term hallucination implies the presence of suffering and distress, which is not always the case here, many authors prefer to call them anomalous experiences (Luhrmann, 2011; Ross, Joshi, & Currie, 1990).

Empirical data from several studies show that mediums (whether linked or not to a religion) have significantly higher dissociation scores than healthy control participants (atheists or religious), but well below the cutoff point for diagnosis of dissociative identity disorders (Moreira‐Almeida, Neto, & Cardeña, 2008; Wahbeh & Radin, 2017). In addition, the relationship between mediumship and possession with past trauma (e.g., physical or sexual abuse in childhood) is not consensual, with some studies indicating higher prevalence of previous traumatic events than controls (Sar, Alioğlu, & Akyüz, 2014), but not others (Maraldi, 2014; Schaffler, Cardeña, Reijman, & Haluza, 2016). Within this context, an important research question is whether pathological and nonpathological dissociation share the same neural and physiological correlates (the same question being valid regarding psychotic‐like experiences and psychotic disorders).

Conventionally, stress is defined as a process arising from real or perceived environmental demands that can be appraised as threatening or harmless, depending on the availability of adaptive coping resources to an individual (McEwen & Gianaros, 2010). The hypothalamic‐pituitary‐adrenal (HPA) axis can be activated by physical stressors (such as exercise or pain), medications, or psychosocial stressors. Evidence indicates that there is great interindividual variability in the response of the HPA axis to stressors (Kudielka & Wüst, 2010) and that, among psychosocial stressors, performing tasks with elements of evaluative threat, uncontrollability, or both, produces the largest and most consistent increases in cortisol (Nicolson, 2008). The Trier Social Stress Test (TSST) is a widespread research protocol for the investigation of physiological and psychological responses to stress in a controlled manner (Kirschbaum, Pirke, & Hellhammer, 1993), it produces a reliable and consistent HPA axis stimulation (Williams, Hagerty, & Brooks, 2004), allowing results to be compared between different studies and populations (Nicolson, 2008). Pathologically dissociated individuals show signs of chronic stress, most studies indicate that they have higher baseline heart rates than healthy controls (Simeon, Yehuda, Knutelska, & Schmeidler, 2008; Wichmann, Kirschbaum, Böhme, & Petrowski, 2017). Their response to an acute stress is blunted and the hypothalamus‐pituitary‐adrenal axis is hypo‐reactive (Wichmann et al., 2017; Zaba et al., 2015).

Regarding stress reactivity in individuals with psychotic disorders, evidence indicates normal responses of anxiety, heart rate, and cortisol levels in chronic disease states (Lange, Deutschenbaur, et al., 2017), but higher anxiety together with a blunted salivary cortisol response in an acute first episode of psychosis and in disease relapses (Lange, Deutschenbaur, et al., 2017; Walter, Fernandez, Snelling, & Barkus, 2018), the same being observed in disease relapses (Lange, Huber, et al., 2017). Regarding nonpathological dissociative experiences, such as mediumship and possession trances, the sparse available evidence indicates a state of physical and mental arousal during the experience (Bastos, Bastos, Dos Santos, et al., 2018; Bastos, Bastos, Osório, et al., 2018; Delorme et al., 2013; Kawai et al., 2001), but exhibiting a flexible autonomic nervous system, as assessed through the heart rate variability method (Bastos, Bastos, Osório, et al., 2018; Seligman & Brown, 2010).

Individuals with acutely or sub‐acutely activated HPA axis tend to have larger pituitary volumes in neuroimaging studies (Axelson et al., 1992; Krishnan et al., 1991). Compared with healthy controls, pituitary gland volume in pathologically dissociated individuals can be greater, as observed in a study on pediatric patients recently diagnosed with posttraumatic stress disorder (Thomas & De Bellis, 2004), or smaller, as noted in a study on adult patients with chronic panic disorder (Kartalci et al., 2011). That seems to depend on the clinical stage of the disease (Pariante, 2008). A similar picture appears in schizophrenia: no difference in pituitary volume was found between established schizophrenia patients and healthy controls (MacMaster et al., 2007; Pariante, 2008), but individuals with an acute first episode of psychosis had larger pituitary volumes (Nordholm et al., 2013).There are no reported studies investigating pituitary volume in nonpathological dissociation.

Individuals with psychotic disorders (schizophrenia) present significantly decreased MLT production, usually associated with circadian and sleep disorders (Bastos et al., 2019). MLT's main metabolite is 6‐sulfatoxymelatonin (aMT6s) (Stehle et al., 2011). aMT6s levels in the first morning urine sample (corrected for urinary creatinine) are strongly correlated with total plasma MLT production during the previous night, as well as with the maximum night MLT values (Cook et al., 2000). Regarding nonpathological dissociation, the only available study on PG and mediumistic experience demonstrated no between‐group differences in the delta of plasma MLT pre‐ and postanomalous occurrence in experienced mediums compared with nonmedium control subjects from the same sociocultural context (Bastos, Bastos, Osório, et al., 2018). In addition to measurements of MLT and its metabolites, the PG volume measured by MRI is also positively related to the degree of PG's activity and functional status (Bumb et al., 2014; Nölte et al., 2009).

Most of the available MRI studies demonstrated smaller PG volume in schizophrenia patients compared with matched healthy controls (Bersani et al., 2002; Fındıklı et al., 2015; Takahashi et al., 2019). With respect to pathologically dissociated individuals, data on PG structure and function is sparse (Bob & Fedor‐Freybergh, 2008). A pathological condition in which dissociation may be present and about which there is some empirical data available on this topic is bipolar affective disorder: affected individuals usually present reduced nocturnal MLT secretion (Nathan, Burrows, & Norman, 1999; Nurnberger et al., 2000), but the gland appears to be anatomically normal (Fındıklı et al., 2015; Sarrazin et al., 2011).

Therefore, to summarize these different lines of evidence, we set the following a priori assumptions:

1. Measuring mental health and anomalous and dissociative experiences in alleged mediums can help to characterize their condition as pathological or nonpathological.
2. The assessment of the PG (and pituitary gland) volume by magnetic resonance imaging in mediums can show if their neuroanatomical correlates are comparable to healthy controls or mentally ill individuals.
3. The determination of the PG baseline secretory activity in mediums can show if their peripheral physiological correlates are comparable to healthy controls or mentally ill individuals.
4. Obtaining additional objective data, in mediums, about the secretory response of the PG both to mediumistic experience and to control tasks can contribute to a greater understanding of the biological mechanisms underlying spiritual experiences.
5. Obtaining additional objective data about the stress response observed in mediums during the spirit possession experience and during control tasks can also help to characterize it, more accurately, as pathological or nonpathological.

Hence, research experiments investigating different aspects of nonpathological dissociative experiences that occur in certain cultural contexts are necessary to help advance our understanding of dissociative phenomena and their mechanisms (Seligman & Brown, 2010). Nevertheless, there is a scarcity of experimental studies trying to understand both the structure and function of the PG in participants with alleged spirit possession. The present study aims to bridge this gap, investigating PG volume and aMT6s levels (reflecting the functional status of PG), as well as pituitary gland volume, in individuals with alleged mediumship, compared with nonmedium control subjects. In addition, it also aims to evaluate urinary aMT6s and stress reactivity in mediums under different conditions.

## 2 METHODS AND RESULTS

This is a comparative experimental study divided in two arms, Experiment 1 and Experiment 2, both conducted in Campo Grande, Brazil, from January 2018 to April 2019. The whole study was approved by the Institutional Review Board of the Federal University of Mato Grosso do Sul (number: 79882117.8.0000.0021) and was carried out in accordance with the International Ethical Guidelines and Declaration of Helsinki.

Experiment 1 consisted of a controlled study evaluating the volume of the PG and the pituitary gland, as well as urinary aMT6s levels in individuals with *psychophonic* mediumship (i.e., speaking under the influence of spirits), compared with control individuals from other religious contexts that do not emphasize dissociative experiences.

In Experiment 2, an experimental research design was applied to study psychophysiological parameters and urinary aMT6s of mediums during the mediumistic experience, comparing with results obtained in the same individuals during the accomplishment of control tasks, out of trance.

The methods and results of Experiment 1 are reported first, followed by methods and results of Experiment 2. The results of both experiments are then discussed.

### 2.1 Experiment 1

#### 2.1.1 Methods

##### Scenery

The followers of Spiritism consider *obsession* as the persistent action that a morally inferior spirit exerts on an incarnated individual. (e.g., taking vengeance on or inflicting pain on him or her). In *disobsession,* mediums enter altered states of consciousness in order to communicate with spirits from the other world, so that these beings, who have done wrong, can be rehabilitated and dissuaded from doing further harm to their victims by a religious leader (who does not go into trance). During the spirit communication, spiritists believe that the medium partly detaches his or her soul from the body, allowing the communicating spirit to utilize the body to manifest itself in the material world either directly (e.g., automatic writing) or through the transmission of thoughts (that can vary from simple sentences to complex narratives) and sensations (Lucchetti et al., 2012; Lucchetti, Lucchetti, Bassi, & Nobre, 2011).

##### Participants

To find qualified mediums (medium group [MG] participants), investigators contacted a regulatory organization for Spiritism in Campo Grande (Spiritist Federation of Mato Grosso do Sul), which indicated five different Spiritist centers where standardized *disobsession* meetings or séances (a type of spirit release therapy) occurred on a weekly basis.

To find participants of similar age for the control group (CG) investigators contacted the Masonic institution “Grande Oriente de Mato Grosso do Sul” (GOMS) in Campo Grande. It has an interreligious character and its affiliated masonic lodges also have female groups (composed of the mason's wives). Study participants were invited sequentially from these institutions.

Only adult females were included in this study. In the MG, only individuals participating as mediums in *disobsession* meetings for 3 years or more were included. Exclusion criteria for both groups were pregnancy, smoking, history of severe traumatic head injury or meningitis, psychiatric illnesses, hypothalamic or pituitary diseases, urinary tract infection, sleep disorders, carriers of cardiac pacemakers, defibrillators, blood vessel clips, prostheses or metal plates, claustrophobia, chronic diseases (e.g., chronic renal failure, lung disease, diabetes mellitus), as well as current use of psychiatric medicines or medications that may interfere with the hormonal analyses (e.g., exogenous MLT).

##### Research procedures

**FIGURE 1:**
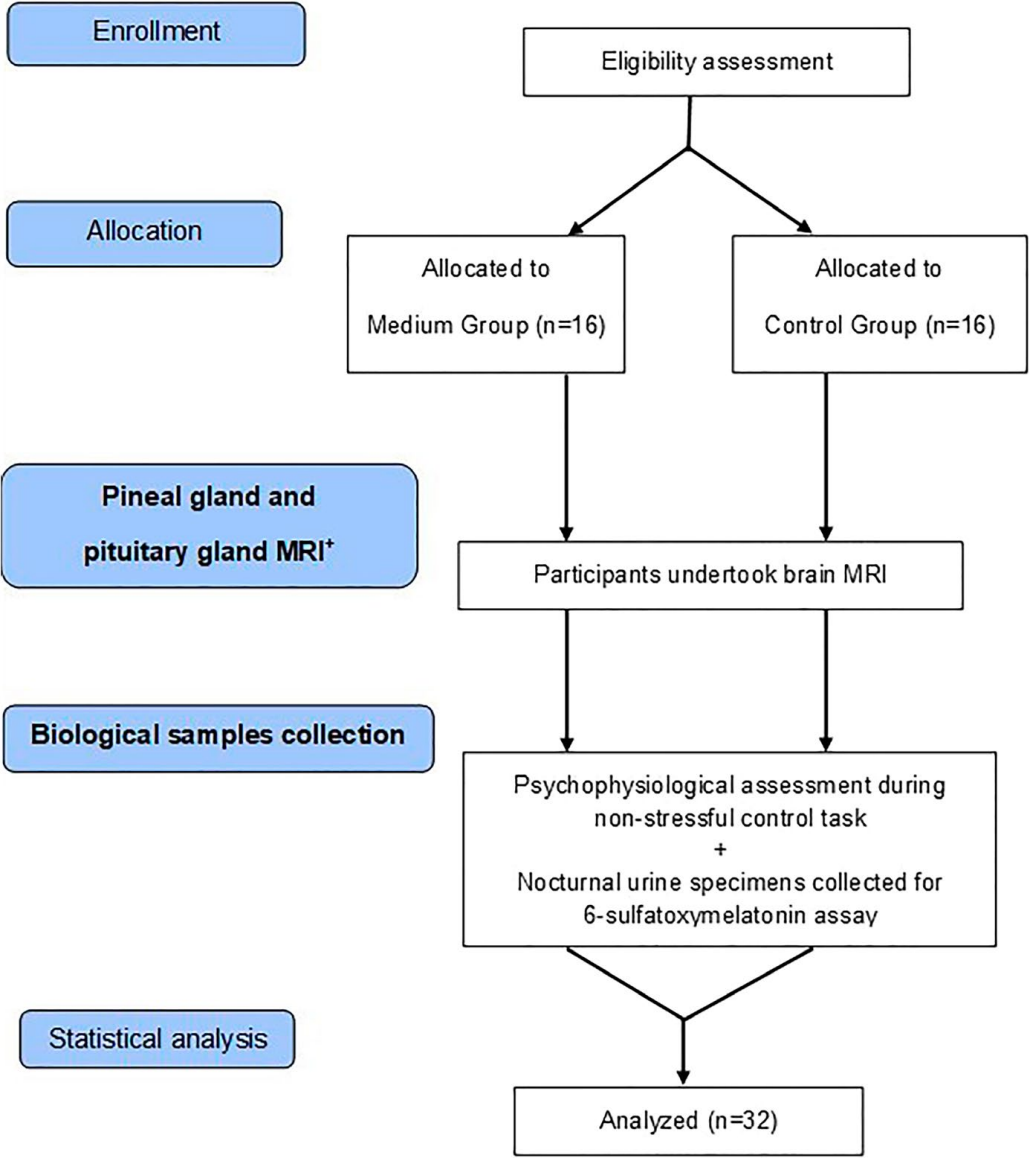
Flowchart illustrating the main steps and procedures of Experiment 1 (^+^magnetic resonance imaging)

The main steps and procedures of Experiment 1 are illustrated by the flowchart in Figure 1 and will be described in detail below.

##### Self‐report measures

Participants answered the following questionnaires prior to the specimens' collection:

###### Mental health

It was assessed with the Self‐Report Psychiatric Screening Questionnaire (SRQ‐20) (Harding et al., 1980), which was validated in Portuguese (Mari & Williams, 1986). This is a 20‐item questionnaire designed to detect common mental disorders in primary care populations; seven or more positive answers suggest a mental disorder.

###### Dissociative experiences scale (DES) (Bernstein & Putnam, 1986)

It was used to screen for dissociative disorders. This instrument was validated in Portuguese (Fiszman, Cabizuca, Lanfredi, & Figueira, 2004; Maraldi & Zangari, 2016) and consists of 28 items related to experiences that one can have in everyday life when not under the influence of psychoactive drugs or alcohol. Some scholars (Carlson et al., 1991; Ross et al., 1990) identify three subscales in DES in addition to the global score: absorption, amnesia, and depersonalization.

###### Anomalous experiences inventory (Menezes, Alminhana, & Moreira‐Almeida, 2012)

This is a 14‐item multiple‐choice instrument where participants report which anomalous experiences (AE) happen frequently to them (Table S1).

###### Pittsburgh sleep quality assessment (PSQI) (Buysse, Reynolds, Monk, Berman, & Kupfer, 1989)

It was used to evaluate the subjective quality of sleep. This instrument was validated in Portuguese (Bertolazi et al., 2011) and consists of 19 items grouped into seven components (Table 1). The higher the score, the worse the quality of sleep.

**TABLE 1:** Psychometric scales scores in the medium group (MG) and control group (CG)

	Group		*p* ^a^	*Effect size* (Cohen's *d*)
	Mediums (*n* = 16)	Controls (*n* = 16)		
	Mean (*SD*)	Mean (*SD*)		
Anomalous experiences	6.1 (2.2)	2.8 (2.4)	<.001*	1.43
SRQ	3.6 (4.1)	5.3 (3.3)	.193	0.46
DES	1.9 (1.2)	1.5 (1.1)	.278	0.35
DES – absorption	32.9 (20.4)	27.2 (18.5)	.601	0.29
DES – depersonalization	11.1 (8.8)	4.6 (6.8)	.060	0.83
DES – amnesia	10.2 (10.0)	9.9 (6.7)	.370	0.03
PSQI	5.9 (3.1)	7.2 (4.0)	.309	0.36
PSQI – Subjective sleep quality	0.8 (0.6)	1.2 (0.8)	.177	0.56
PSQI – Sleep latency	1.1 (0.8)	1.6 (1.0)	.138	0.55
PSQI – Sleep duration	0.9 (0.9)	1.1 (0.9)	.268	0.22
PSQI – Sleep efficiency	0.4 (0.9)	0.4 (0.8)	.436	0.0
PSQI – Sleep disturbance	1.4 (0.6)	1.7 (0.7)	.236	0.46
PSQI – Use of sleep medication	0.2 (0.5)	0.4 (1.0)	.370	0.25
PSQI – Daytime dysfunction	1.1 (0.8)	0.8 (0.8)	.398	0.37

Abbreviations: DES, Dissociative Experiences Scale; PSQI, Pittsburgh Sleep Quality Index; *SD*, Standard deviation; SRQ, Self‐Report Psychiatric Screening Questionnaire.

^a^ Student's test.

* *p* < .05.

###### Questionnaire on mediumship (adapted from Negro, Palladino‐Negro, & Louzã, 2002)

It was answered by MG participants, consisting of simple questions to assess their degree of training, frequency, and nature of mediumship behavior. At the end of the experiment, they were also asked to fill out a visual analogue self‐report scale (10 cm line, labelled from 0 to 10) to rate the extent to which experimental procedures had any influence on their spirit communication, with 0 meaning no influence at all and 10 meaning a very serious influence.

##### Procedures for evaluation of the pineal gland (PG) and pituitary gland by magnetic resonance imaging (MRI)

Noncontrast MRI scans were performed using a 3T MR scanner (Phillips MR Systems Achieva) at the Pró‐Exames Clinic (Campo Grande). The T1‐weighted, 3‐dimensional, fast field echo (FFE) technique data set consisted of 320 sagittal sections (section thickness, 1 mm; voxel size, 1 × 1 × 1 mm; field of view, 256 × 256 mm^2^; repetition time, 3,000 ms; echo time, 3.7 ms; flip angle, 8°). The T2‐weighted, 3‐dimensional, spin‐echo (SE) technique data set consisted of 381 sagittal sections (section thickness, 1 mm; voxel size, 1 × 1 × 1 mm; field of view, 256 × 256 mm^2^; repetition time, 3,100 ms; echo time, 245 ms; flip angle, 90°). The total acquisition time of the exam was 9 min.

An experienced biomedical and MRI technician, blinded to the group in which the participant was allocated, performed the analysis of the exams. To determine interexaminer variability, a second practitioner (certified neuroradiologist), also blinded as to the participant's group and to the results of measurements by the first practitioner, assessed the data set of 10 randomly picked participants. Before the scans, each participant also had their weight, height and cephalic perimeter measured. In addition, participants were also asked about handedness and frequency of alcohol consumption, as these variables might influence the measurements of brain volumes and areas.

The PG volume was determined by manually defining the margins that limit the gland, using T1‐weighted images. Estimation of gland volume was performed using the ellipsoid formula, directly measuring the maximum parameters in two‐dimensional (2D) images (volume = length × width × height/2) (Bersani et al., 2002; Fındıklı et al., 2015).

Similarly, the pituitary gland volume was determined by manually defining the margins that limit the gland, using T1‐weighted images. Estimation of gland volume was performed using the same aforementioned ellipsoid formula (Naik, Reddy, Srinath, & Kumar, 2015). As standardized in the literature, the bright segment corresponding to the neurohypophysis was included in the measurement, but the pituitary stem was excluded. As an individual's total brain volume may influence volumetric measurements of intracerebral structures of interest, experts suggest that structural neuroimaging studies always control for this variable (Anstey et al., 2007; Nordenskjöld et al., 2013). However, currently, MRI brain volume measurement techniques require laborious and time‐consuming manual segmentation procedures (Ambarki, Wåhlin, Birgander, Eklund, & Malm, 2011; Nordenskjöld et al., 2013). For this purpose, the determination of intracranial area has been considered a simple but reliable alternative method (Ferguson, Wardlaw, Edmond, Deary, & Maclullich, 2005; Nandigam et al., 2007; Piper, Yoong, Pujar, & Chin, 2014). Therefore, the mid‐sagittal intracranial area was an additional neuro‐morphometric parameter that was measured in all participants.

##### Urine sample collection procedures

Seeking greater collection conditions homogeneity between groups, the urine samples collections for the aMT6s assay (pool sample from 23:00 to 06:00 the following morning) were performed on nights in which participants performed a *nonstressful control task*. An adapted version of the *control protocol* for the TSST (Von Dawans, Kirschbaum, & Heinrichs, 2011), was applied. The main activity in this task consisted of group reading (in a meeting with 2–6 participants, usually all acquainted). The text chosen for reading by all participants was the moral tale: “The Three Questions,” by Leo Tolstoy (available on: https://www.plough.com/en/topics/culture/short‐stories/the‐three‐questions).

For the MG, the nonstressful control task was performed in the Spiritist center that the participant attends, and for the CG, it was performed in the masonic institution that the participant attends. The urine samples were collected by the participants themselves at home. The samples were then stored at −20°C in Laboratório Célula (Campo Grande) until the performance of the aMT6s assay. All laboratory personnel remained blinded to the group allocation of participants.

###### Urinary 6‐sulfatoxymelatonin (aMT6s) assay

On the urine collection nights, all participants were asked to go to bed at their homes at 23:00 hr, avoiding exposure to light at 23:00 hr to 06:00 the next morning. The literature recommends a correction of aMT6s by urinary creatinine, which significantly improves the correlation with plasma MLT (Cook et al., 2000). Thus, creatinine was assayed in the urine samples through kinetic method using a commercial kit (Hitachi Cobas c311; Roche Diagnostics) at the Laboratório Célula (Campo Grande). Subsequently, all aMT6s analyzes were performed at the Molecular Biology Laboratory of the Federal University of Mato Grosso do Sul (Campo Grande). To quantify the aMT6s levels, the ELISA method (enzyme‐linked immunosorbent assay) was used (IBL). The absorbances of the samples were read by the microtiter plate reader Molecular Devices SpectraMax 190^®^, at 450 nm. The sensitivity of the assay was 1.0 ng/ml, with maximal intra‐ and interassay coefficients of variation (CV) of 12.2% and 14.9%, respectively.

##### Measurement of psychophysiological parameters during the nonstressful task

As recommended by the aforementioned *control protocol* for the TSST (Von Dawans et al., 2011), subjective and objective measures of stress response were undertaken throughout the task. Saliva samples were collected for free cortisol assaying in 4 time points: baseline (10 min after the subject arrived at the site), immediately before the activity begins (at the end of an introductory and explanatory phase of the task), postactivity (2 min after the end of the group reading phase) and on recovery (35 min after the end of the group reading phase). At each of these 4 time points, participants were asked to fill in the STAI‐S‐6 instrument and their heart rate was measured.

###### Anxiety

The anxiety state was assessed with the State‐Trait Anxiety Inventory (STAI) (Spielberger, Gorsuch, & Lushene, 1970). In the present study, a short‐form of the STAI‐State scale (STAI‐S‐6) (Marteau & Bekker, 1992), which was validated in Brazilian Portuguese (Fioravanti‐Bastos, Cheniaux, & Landeira‐Fernandez, 2011), was used.

###### Heart rate

Measurements of heart rate were performed with a pulse oximeter (SB 100, Rossmax), by placing a sensor in a chirodactyl.

###### Salivary cortisol

Saliva samples were collected using Salivette^®^ tubes (Sardstedt). In the Laboratório Célula (Campo Grande), the samples were centrifuged, and their cortisol concentrations were determined by electrochemiluminescence (Hitachi Cobas e411 equipment; Roche Diagnostics). The sensitivity of the salivary cortisol assay was 0.16 µg/dl, with maximal intra‐ and interassay CV of 8.6% and 8.7% respectively.

##### Statistical analysis

Statistical power calculations were performed based on previous studies evaluating PG volume (Bumb et al., 2014; Sarrazin et al., 2011) and urinary aMT6s (Crasson et al., 2004; Santoro, Giacheti, Rossi, Campos, & Pinato, 2016) under different clinical conditions. Assuming *α* = .05 and 1 – *β* = .95, regarding PG volume, a sample size of 15 participants was estimated and, regarding aMT6s, 12 participants were estimated. In order to compensate for possible losses to follow‐up, a target sample size of 16 participants in each group was adopted. Collected data were entered into the SPSS 20.0 statistics package (IBM Corporation). Logarithmic transformation was performed on all continuous variables before analysis for normalization of variables. Demographic, psychometric, laboratory, and imaging data were analyzed using chi‐square test or Fisher's exact test (for categorical variables) and Student's *t* test (for continuous variables). For between‐group comparisons (MG vs. CG) of continuous variables, independent *t* tests were performed. For within‐group comparisons regarding time factor, linear model repeated measures ANOVA tests with Bonferroni post hoc tests were performed. Data were expressed as mean ± standard deviation and percentages. Differences were considered statistically significant when *p* < .05. Effect sizes (Cohen's *d*) were calculated for the results of each between‐group comparison using an online calculator (https://www.socscistatistics.com/effectsize/default3.aspx). The cutoff points for interpreting effect sizes were small (0.2), medium (0.5), and large (0.8). Spearman correlation tests were performed to investigate possible relationships between variables considered relevant.

#### 2.1.2 Results

##### Between‐group comparisons regarding sociodemographic and anthropometric aspects

Thirty‐two women (16 in MG and 16 in CG) participated in this study. There were no between‐group differences regarding age (MG 55.4 ± 8.9 vs. CG 51.2 ± 7.5, *p* = .16), self‐reported ethnicity, or education. Between‐group differences were noted for marital status, more CG participants were married, and religion, all participants in the MG were Spiritists, while all participants in the CG were non‐Spiritists (15 catholic and one gnostic). Table S2 depicts the results of sociodemographic data of the 32 participants.

##### Between‐group comparisons on self‐reporting instruments

Subjects in the MG had volunteered as mediums at the religious centers for a long period of time (22.3 ± 11.0 years), most (69%) had acted as mediums for more than 10 years. Most of the mediums (75%) described full consciousness during *psychophony*, whereas some (25%) reported partial consciousness, and none described complete lack of awareness. All subjects reported being in control of the mediumship phenomenon; half reported always being in complete control, and half reported to be frequently in control of the experience. Most of mediums (94%) reported that they had formal mediumship training, which consisted of religious courses (weekly meetings and supervision for 1 year or more). Most of the subjects in the MG reported little influence of the experiment procedures on their spirit communication (0–10 scale: 0.3 ± 0.4).

Subjects in the MG reported a significantly higher number of anomalous experiences than the subjects in the CG (Table 1). The following individual anomalous experiences were reported with statistically higher frequency by MG participants: apparitional experiences, spiritual perception, abnormal dreams, possession, and psychography (Table S1).

No significant differences in subjects’ mental health were noted between the groups, as measured by the SRQ instrument (Table 1). The mean scores on this scale for both groups were lower than the cutoff (endorsement of seven or more items), indicating low probability for developing common mental disorders.

No significant differences in the total score of dissociative experiences (DES) were noted between the groups (Table 1). The mean scores on this scale for both groups were well below the cutoff (score of 30 or more), arguing against the presence of dissociative disorders. Regarding the independent evaluation of the scores in each subscale (absorption, depersonalization, and amnesia), no between‐group differences were detected for absorption or amnesia. However, a large effect size (*d* = 0.826) was observed for higher depersonalization scores in the MG, though not reaching statistical significance (*p* = .060) (Table 1).

Sleep quality was similar between MG and CG, as measured by global scores in the PSQI instrument. In addition, for both groups, the mean overall scores exceeded the cutoff (>5), suggesting that the subjects were equally “bad sleepers” (Table 1).

Since there was a difference between the groups regarding demographic aspects “marital status” and “religion,” we investigated possible correlations of these variables with scores on self‐report instruments in the total sample. Regarding marital status, no significant correlations were noted. Regarding religion, the “Spiritist” religion was positively correlated with the number of anomalous experiences and with depersonalization scores, but inversely correlated with the scores of mental health problems (SRQ) (Table S3).

##### Between‐group comparisons of structural brain magnetic resonance imaging (MRI) parameters

Regarding the anthropometric variables that were measured in order to control for a possible influence over the measurements of volumes of specific intracerebral structures, no between‐group differences were noted for BMI or cephalic perimeter (Table S4). Similarly, there was no difference between groups regarding dominance. However, MG participants reported a significantly lower frequency of alcohol consumption than CG participants (*p* = .012) (Table S5).

**FIGURE 2:**
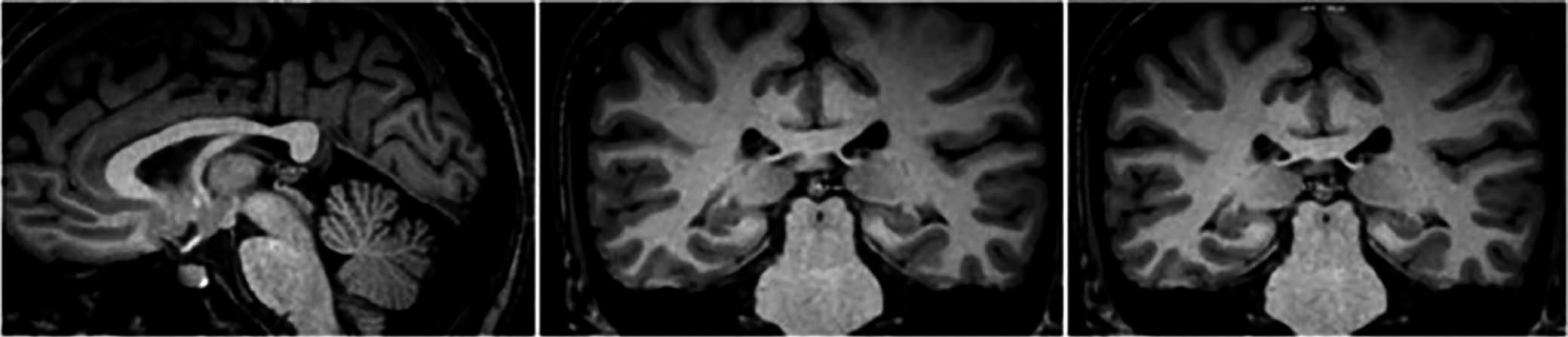
Images exemplifying magnetic resonance (MR) measurement of the research participant's pineal gland (PG) volume

**FIGURE 3:**
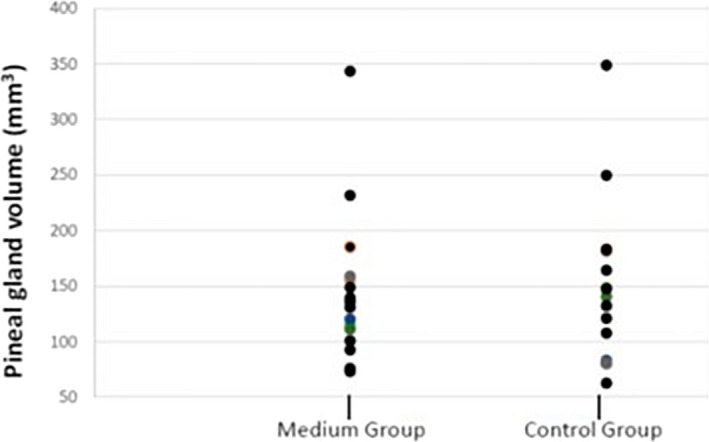
No differences in pineal gland (PG) volume between medium group (MG) and control group (CG)

No differences were found between groups for any of the structural brain MRI parameters: mid‐sagittal intracranial area (MG 14,127.1 ± 1,064.7 vs. CG 13,651.1 ± 1,099.0 cm^2^, *p* = .219, *d* = 0.44) (Figures S1 and S2); pineal gland volume (MG 144.6 ± 66.6 vs. CG 150.7 ± 72.1 cm^3^, *p* = .873, *d* = 0.09) (Figures 2 and 3) and pituitary gland volume (MG 578.1 ± 181.6 vs. CG 618.2 ± 162.9 cm^3^, *p* = .418, *d* = 0.23 [Figures S3 and S4]). As there were no significant differences between the groups for mid‐sagittal intracranial area, we considered that individual differences in this parameter did not influence the measurements of the intracerebral structures of interest. Therefore, for the final analysis, we did not use corrected values but the absolute values of measurements for each structure.

Calculations of the correlations between the structural neuroimaging parameter measurements made by the two collaborating research experts indicate that there were high agreement and low interexaminer variability (Table S6).

Since the groups were different regarding the frequency of alcohol consumption, we investigated possible correlations of this variable with the parameters of neuroimaging in the total sample. The only significant relationship found was a direct correlation between frequency of alcohol consumption and pituitary gland volume in the CG (*r* = .51, *p* = .042) (Table S7).

##### Between‐group comparison of urinary 6‐sulfatoxymelatonin (aMT6s) concentrations in samples collected after nonstressful control task (NS)

No differences were found between MG and CG regarding creatinine‐corrected urinary aMT6s levels collected on nights when participants performed the NS (Table 2). The measurement of psychophysiological parameters during the performance of the nonstressful task points to an equivalence in the collection conditions, no significant differences were observed between the groups for any of these parameters (Table 2, Figure S5).

**TABLE 2:** Comparison of urinary 6‐sulfatoxymelatonin concentrations and psychophysiological parameters at night of sample collection (nonstressful control task) between medium (MG) and control group (CG)

	Group		*p* ^a^	Effect size (Cohen's *d*)
	Mediums (*n* = 16)	Controls (*n* = 16)		
	Mean (*SD*)	Mean (*SD*)		
NS – sulfatoxyMLT	51.8 (44.8)	55.5 (31.6)	.280	0.418
STAI‐S T1	35.2 (11.4)	35.2 (10.9)	.990	0.0
STAI‐S T2	31.9 (12.2)	34.2 (13.4)	.649	0.179
STAI‐S T3	29.8 (8.1)	32.7 (12.2)	.099	0.280
STAI‐S T4	27.5 (9.8)	31.2 (10.2)	.273	0.370
HR – T1	76.2 (12.5)	75.8 (7.8)	.978	0.038
HR – T2	74.4 (9.8)	72.6 (7.1)	.640	0.210
HR – T3	70.4 (9.7)	74.4 (8.6)	.228	0.436
HR – T4	70.2 (9.4)	73.1 (8.9)	.368	0.316
Cortisol (µg/dl) – T1	0.15 (0.0)	0.17 (0.1)	.139	0.283
Cortisol – T2	0.15 (0.0)	0.18 (0.1)	.120	0.424
Cortisol – T3	0.16 (0.0)	0.17 (0.1)	.388	0.141
Cortisol – T4	0.15 (0.0)	0.17 (0.1)	.139	0.283

Note

No between‐group differences.

Abbreviations: HR, Heart rate (bpm); NS, Nonstressful control task; *SD*, Standard deviation; STAI‐S State‐Trait Anxiety Inventory – state component, short‐form; sulfatoxyMLT, Creatinine‐corrected 6‐sulfatoxymelatonin (ng/g creatinine); T, Time point.

^a^ Student's *t* test.

* *p* < .05.

However, the within‐group comparison (repeated measures ANOVA with factor “time”) of the psychophysiological parameters “heart rate” and “anxiety state” along the nonstressful control task (assessed in 4 time points) showed that the mental and physical relaxation response was more significant in the MG than in the CG (Table 3).

**TABLE 3:** Multiple within‐group comparisons of psychophysiological parameters at each time point (T) during nonstressful task control. Significant reduction in heart rate (within‐group) noted only in medium group

	Mediums (*n* = 16)				*p* ^a^	Controls (*n* = 16)				*p* ^a^
	T1^(s)^	T2^(t)^	T3^(u)^	T4^(v)^		T1^(w)^	T2^(x)^	T3^(y)^	T4^(z)^	
	Mean (*SD*)	Mean (*SD*)	Mean (*SD*)	Mean (*SD*)		Mean (*SD*)	Mean (*SD*)	Mean (*SD*)	Mean (*SD*)	
STAI‐S	35.2 (11.4)	31.9 (12.2)	29.8 (8.1)	27.5 (9.8)	.059^b^	35.2 (10.9)	34.2 (13.4)	32.7 (12.2)	31.2 (10.2)	.124
HR	76.2 (12.5)	74.4 (9.8)	70.4 (9.7)	70.2 (9.4)	.007^c^	75.8 (7.8)	72.6 (7.1)	74.4 (8.6)	73.1 (8.9)	.462
Cortisol (µg/dl)	0.15 (0.0)	0.15 (0.0)	0.16 (0.0)	0.15 (0.0)	.299	0.17 (0.1)	0.18 (0.1)	0.17 (0.1)	0.17 (0.1)	.552

Abbreviations: HR, Heart rate (bpm); *SD*, Standard deviation; STAI‐S, State‐Trait Anxiety Inventory – state component, short‐form.

^a^ Repeated measures ANOVA.

^b^ Post hoc comparisons: s > v (*p* = .027).

^c^ Post hoc comparisons: s > v (*p* = .017) and t > v (*p* = .050).

Since the groups differed regarding the frequency of alcohol consumption, we investigated a possible correlation of this variable with the concentrations of corrected aMT6s. In addition, we also investigated possible correlations of this hormone levels with some other variables that we considered physiologically relevant: age, PG volume, and sleep quality (global scores on the PSQI). The only significant relationship found was a positive correlation between aMT6s levels and PG volume in MG (*r* = .61, *p* = .012) (Table S8).

### 2.2 Experiment 2

#### 2.2.1 Methods

##### Participants

Participants enrolled for Experiment 2 were the same as those enrolled for Experiment 1, but only those from the MG. Moreover, only participants with complete results for all study parameters were included in final analysis of Experiment 2.

##### Procedures

Psychophysiological measurements were undertaken in MG during mediumistic experience. Furthermore, participants returned at the same daytime (19:00–21:00) and place where they usually manifest mediumistic experiences, on other days, to perform control tasks: a nonstressful control task and a stressful control task, both out of trance. On each evening, participants collected urine samples at their homes (pool sample from 23:00 to 06:00 the following morning) for the aMT6s assay.

**FIGURE 4:**
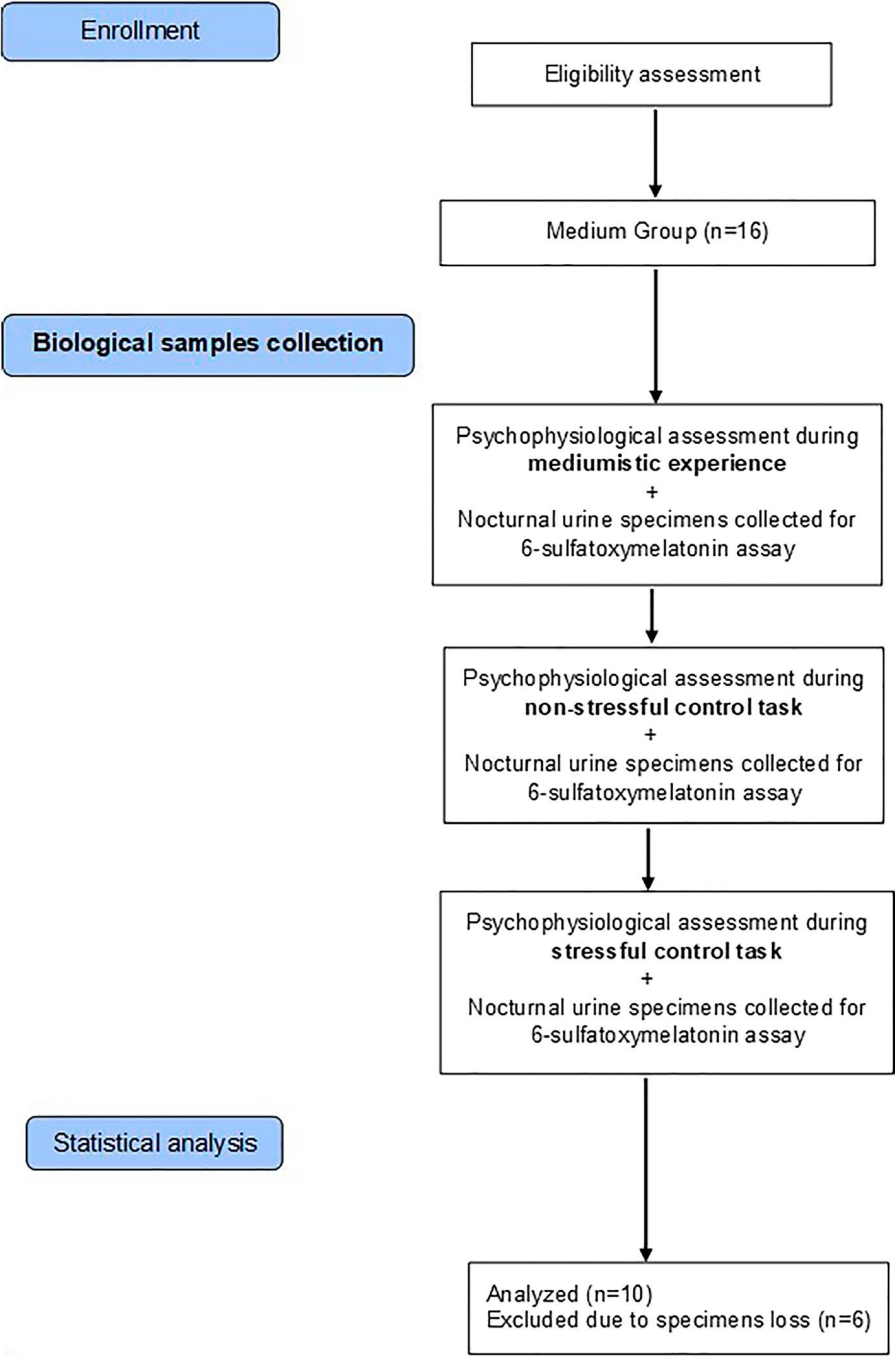
Flowchart illustrating the main steps and procedures of Experiment 2

The main steps and procedures of Experiment 2 are illustrated by the flowchart in Figure 4 and will be described in detail below.

###### Mediumistic experience (M)

A naturalistic approach was adopted. The experimental mediumistic meetings took place at the Spiritist centers that the participants usually attended, with the staff they were used to work with, keeping the routine format and times of the meeting.

###### Nonstressful control task (NS)

This task consisted of reading, in group, a text (shorty) with moral background. Details regarding the planning and execution of the nonstressful control task have already been described for Experiment 1 (see Section 2.1.1.6).

###### Stressful control task (S)

The TSST was applied to induce acute laboratory stress. This paradigm consists of giving a speech and a mental calculation task in front of an evaluative committee. The TSST reliably induces subjective stress with concomitant release of cortisol, which normally rises 2–4 times above baseline levels (Kirschbaum et al., 1993). In the present study, as we evaluated two participants on each occasion (both members of the MG), we used the group version of the TSST (TSST‐G) (Childs, Vicini, & De Wit, 2006). Until the end of the recovery phase, participants were kept in separate rooms with minimal verbal or visual contact between each other. The TSSTs were performed at the Endocrinology Clinic “Endo Síntese” (Campo Grande).

##### Detail on the execution of Experiment 2

During M, NS, and S, saliva samples were collected for free cortisol assaying in 4 time points: baseline (10 min after the subject arrived at the site); immediately before the activity begins (immediately before the mediumistic communication phase of the mediumistic meeting begins and at the end of the preparation phase of NS and S); postactivity (2 min after the end of the first mediumistic communication performed by the medium being studied, and 2 min after the end of the stressful and nonstressful control tasks); and on recovery (35 min after the end of the last mediumistic communication performed by the medium being studied, and 35 min after the end of S and NS). At each of these 4 time points, participants were asked to fill in the STAI‐S‐6 instrument, and their heart rate was measured.

The procedures adopted for the urinary aMT6s assay and to evaluate subjective and objective stress response have already been described in the corresponding sections of Experiment 1 (see Sections 2.1.1.5 and 2.1.1.6). In general, the activities were performed with an interval of 1 week between each event.

##### Statistical analyses

The procedures adopted for the Statistical analyses have already been described in the corresponding section of Experiment 1 (see Section 2.1.1.7).

#### 2.2.2 Results

There was improper disposal of participants' urine samples in the laboratory where the samples were being stored frozen. Upon this loss, only 10 mediums had complete results. Therefore, the sample included for final analysis in Experiment 2 consisted of 10 participants.

The sample from Experiment 2 had a mean age of 57.8 ± 9.5, a number of reported anomalous experiences of 5.6 ± 2.2, mental health score (SRQ) of 3.2 ± 3.2, a subjective quality of sleep score (PSQI) of 5.8 ± 3.2, and had volunteered as mediums in Spiritist centers for 25.8 ± 10.2 years.

##### Within‐group comparisons in the medium group (MG)—time effect

During the nonstressful control task, a significant difference was noted along the task only for heart rate: there was a reduction in HR along the execution of the task. No significant differences were noted along the task for “anxiety state” or for salivary cortisol (Table 4).

**TABLE 4:** Multiple comparisons in the medium group (*n* = 10) regarding psychophysiological parameters during nonstressful control task, mediumistic experience and stressful control task (time effect)

	T1^(s)^	T2^(t)^	T3^(u)^	T4^(v)^	*p* ^a^
	Mean (SP)	Mean (*SD*)	Mean (*SD*)	Mean (*SD*)	
Nonstressful control task (NS)					
STAI‐S	39.0 (11.4)	34.0 (14.6)	31.0 (9.0)	30.0 (11.4)	.131
HR	71.3 (8.6)	71.1 (9.6)	65.4 (7.7)	65.7 (8.1)	.029^b^
Cortisol (µg/dl)	0.15 (0.0)	0.15 (0.0)	0.15 (0.0)	0.15 (0.0)	.343
Mediumistic experience (M)					
STAI‐S	35.0 (9.0)	38.7 (11.5)	41.0 (14.4)	29.7 (8.8)	.184
HR	71.5 (10.5)	73.3 (12.7)	77.5 (14.1)	71.1 (9.4)	.144
Cortisol	0.15 (0.0)	0.15 (0.0)	0.15 (0.0)	0.16 (0.0)	.343
Stressful control task (S)					
STAI‐S	36.3 (12.7)	48.7 (16.9)	51.3 (13.4)	33.0 (13.2)	.021^c^
HR	72.1 (9.2)	76.3 (8.6)	75.5 (10.8)	66.9 (8.5)	.007^d^
Cortisol	0.15 (0.0)	0.15 (0.0)	0.16 (0.0)	0.26 (0.2)	.093

Abbreviations: HR, Heart rate (bpm); *SD*, Standard deviation; STAI‐S, State‐Trait Anxiety Inventory – state component, short‐form.

^a^ Repeated measures ANOVA.

^b^ Post hoc comparisons: s > u (*p* = .031); s > v (*p* = .014); t > v (*p* = .049).

^c^ Post hoc comparisons: s < t (*p* = .026); s < u (*p* = .049) and u > v (*p* = .008).

^d^ Post hoc comparisons: s > v (*p* = .039) and t > v (*p* = .002).

During the mediumistic experience, no significant differences were noted along the experience for any of the three types of psychophysiological measurements (Table 4).

During the stressful control task, a significant difference was noted along the task for “anxiety state” and for “heart rate.” For both variables, along the task, there was an initial increase followed by a reduction in mean values measured (Table 4).

##### Within‐group comparisons in the medium group (MG) ‐ condition effect

No significant differences were found regarding nocturnal urinary aMT6s levels in mediums between the three different conditions (NS, M, and S) (Table 5).

**TABLE 5:** Multiple comparisons in the medium group (*n* = 10) regarding psychophysiological parameters and urinary 6‐sulfatoxymelatonin during nonstressful control task (NS), mediumistic experience (M), and stressful control task (S)—condition effect

	Condition			*p* ^a^
	NS	M	S	
	Mean (*SD*)	Mean (*SD*)	Mean (*SD*)	
STAI‐S T1	39.0 (11.4)	35.0 (9.0)	36.3 (12.7)	.481
STAI‐S T2	34.0 (14.6)	38.7 (11.5)	48.7 (16.9)	.055
STAI‐S T3	31.0 (9.0)	41.0 (14.4)	51.3 (13.4)	.005^b^
STAI‐S T4	30.0 (11.4)	29.7 (8.8)	33.0 (13.2)	.650
HR T1	71.3 (8.6)	71.5 (10.5)	72.1 (9.2)	.939
HR T2	71.1 (9.6)	73.3 (12.7)	76.3 (8.6)	.240
HR T3	65.4 (7.7)	77.5 (14.1)	75.5 (10.8)	.008^c^
HR T4	65.7 (8.1)	71.1 (9.4)	66.9 (8.5)	.089
Cortisol (µg/dl) T1	0.15 (0.0)	0.15 (0.0)	0.15 (0.0)	.343
Cortisol T2	0.15 (0.0)	0.15 (0.0)	0.15 (0.0)	>.999
Cortisol T3	0.15 (0.0)	0.15 (0.0)	0.16 (0.0)	.367
Cortisol T4	0.15 (0.0)	0.16 (0.0)	0.26 (0.2)	.201
SulfatoxyMLT	37.3 (24.1)	48.0 (44.2)	43.2 (21.5)	.600

Abbreviations: HR, Heart rate (bpm); *SD*, Standard deviation; STAI‐S, State‐Trait Anxiety Inventory – state component, short‐form anxiety; SulfatoxyMLT, Creatinine‐corrected 6‐sulfatoxymelatonin in urine (ng/g); T, Time point.

^a^ Repeated measures ANOVA.

^b^ post hoc comparisons: NS < S (*p* = .004) and M < S (*p* = .046).

^c^ post hoc comparisons: NS < M (*p* = .015).

When comparing psychophysiological parameters of mediums between the three different conditions, differences were found for anxiety state and heart rate only at time point 3 (reflecting the time immediately after group reading in NS activity, the time immediately after mediumistic communication in M activity, and the time immediately after the interview in S activity (Table 5). The anxiety scores at time point 3 in the S condition were higher than those in the NS and M conditions (Table 5). For heart rate, mean values at time point 3 in the M condition were greater than those in the NE (Table 5). For salivary cortisol, no significant differences were found between the three different conditions at any time point of measurement (Table 5).

**FIGURE 5:**
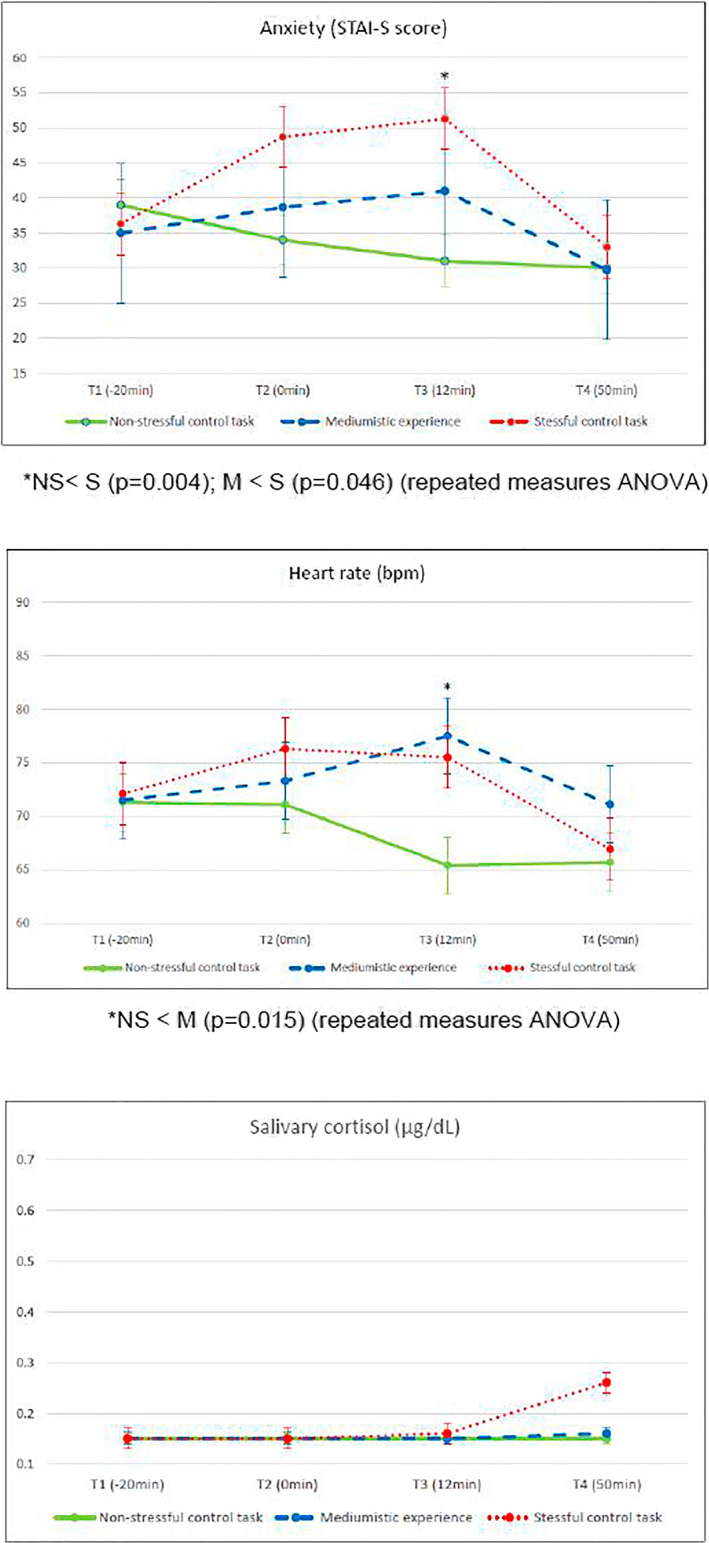
Psychophysiological parameters in the medium group (*n* = 10) during nonstressful control task (NE), psychic experience (M) and stressful control task (E). Point represents the mean and vertical bar the standard error. Significant differences indicated in the graphs of each parameter

Figure 5 illustrates the results of psychophysiological parameter measurements in the MG during NS, M, and S.

## 3 DISCUSSION

### 3.1 Self‐report measures

Despite mediums presented significantly higher scores for anomalous experiences and for dissociative experiences (depersonalization subscale), we found no between‐group differences regarding mental health scores (SRQ). Mediums in our sample presented mean total dissociation scores well below the diagnostic cutoff point for dissociative identity disorder (Bernstein & Putnam, 1986), which corroborates previous studies on mediumship (Moreira‐Almeida et al., 2008; Vencio, Caiado‐Vencio, & Caixeta, 2019; Wahbeh & Radin, 2017). These data are in line with recent research suggesting that mediums are socially well‐adapted and occupationally active (Moreira‐Almeida et al., 2008; Negro et al., 2002) reinforcing the proposition of mediumship as a nonpathological dissociative phenomenon.

### 3.2 Structural imaging and baseline secretory activity of the pineal gland (PG)

In the present study, no differences were found between the MG and the nonmedium CG in terms of PG volume, nocturnal urinary aMT6s levels, and self‐reported subjective sleep quality (PSQI). In other words, structurally and functionally normal PGs were found in mediums, that are individuals often considered to have psychotic‐like experiences. This picture contrasts sharply with the data available in the literature describing these aspects in individuals with psychotic disorder/schizophrenia. In patients with schizophrenia, there is a predominantly reduced PG volume (Bersani et al., 2002; Fındıklı et al., 2015; Takahashi et al., 2019), reduced MLT production and higher frequency of sleep disturbance, when compared to healthy controls (for review, see, e.g., Bastos et al., 2019).

### 3.3 Structural imaging of pituitary gland

The pituitary gland volume was similar between mediums and controls, in contrast to the pattern observed in individuals at high risk or in an acute episode of psychosis, who present higher pituitary volume (generally explained as being a reflex of the acute activation of the hypothalamus‐pituitary‐adrenal axis that occurs in this situation) (Nordholm et al., 2013; Pariante, 2008; Pariante et al., 2004).

### 3.4 Assessment of stress response during spirit possession and control tasks

Measurements of psychophysiological parameters (subjective anxiety state, HR, and salivary cortisol) (Experiment 2) in MG, on nights when they had mediumistic communication, showed an arousal response of low intensity, without statistically significant intra‐individual differences. These data corroborate the recent works (Beischel, Tassone, & Boccuzzi, 2019; Wahbeh, Cannard, Okonsky, & Delorme, 2019) which found no significant intra‐individual differences in mediums during the anomalous experience regarding various physiological parameters either. However, other previous works demonstrated a pattern of significant sympathetic activation in response to the experience (Bastos, Bastos, Osório, et al., 2018; Kawai et al., 2001). The phenomenon of stress habituation should be kept in mind when interpreting these findings. Experienced mediums may show an already attenuated stress response triggered by mediumistic experience. Heart rate variability data from a previous study with a similar population reinforce this explanation: correlational data demonstrated that the more experienced the medium, the lower the physical arousal in response to the mediumistic experience ‐ suggesting stress habituation (Bastos, Bastos, Osório, et al., 2018).

The comparison of psychophysiological parameters (mainly anxiety state and HR), in mediums, between the three different conditions (nonstress control task, mediumistic experience, and stress control task) indicated that the degree of mental and physical arousal in response to the mediumistic practice was intermediate between the three conditions. The pattern of stress reactivity that is reported in individuals with pathological dissociation (e.g., posttraumatic stress disorder) is one in which subjective and objective anxiety parameters rise rapidly with the onset of the induced stress, remains elevated throughout the test and do not decrease after the stress termination (Giesbrecht, Merckelbach, van Oorsouw, & Simeon, 2010). Conversely, in the present study, MG's subjective anxiety and HR levels decreased rapidly after the stress termination, indicating an efficient emotion regulation. These findings are congruent with HRV data from previous studies with mediums indicating they have a flexible autonomic nervous system (Bastos, Bastos, Osório, et al., 2018; Seligman & Brown, 2010).

Despite the changes that were observed in anxiety state and in HR, the salivary cortisol response, on both nights of mediumistic experience and of stressful control task (TSST), did not show significant variations. We identify some factors that may explain this finding, which will be discussed as follows. The standard recommendation of TSST developers is that the test should be performed in the afternoon, when there is greater stability of the HHA axis (Kirschbaum et al., 1993). In the present study, we chose to apply it at night because a naturalistic approach was adopted, comparing it with the mediumistic experience, which usually occurs at night in Spiritist centers. This may have influenced our results, but there is also evidence indicating that TSST responses in salivary and plasma cortisol are comparable for when testing occurred at different times of the day between 09:00 am and 7:00 pm (Kudielka, Schommer, Hellhammer, & Kirschbaum, 2004). In our study, another aspect that may have contributed to the pattern of attenuated cortisol response during TSST is that the members of the TSST interview panel were female, as were the participants. Duchesne, Tessera, Dedovic, Engert, and Pruessner (2012) showed that, in young men and women, a cortisol increase was noted only when the panel members were of the opposite sex. To our knowledge, no prior studies have examined whether this also occurs in mature participants, but this methodological aspect may have influenced our results. Additionally, in women of childbearing age, the stage of the menstrual cycle may influence the response of the corticotrophic axis to the TSST (smaller cortisol response during the follicular stage) (Allen, Kennedy, Cryan, Dinan, & Clarke, 2014). However, in our sample, we believe that this might have not been an issue because most participants were postmenopausal. Two other factors that may also have contributed to the attenuated cortisol response in our sample are as follows: the availability of social support and the frequent meditative/contemplative practice, both having been linked to decreased cortisol response to stress (Eisenberger, Taylor, Gable, Hilmert, & Lieberman, 2007; Kemeny et al., 2012) and both strongly present in the context of Spiritist mediumship.

### 3.5 Pineal gland secretory activity after spirit possession and control tasks

Regarding the comparison of nocturnal urinary aMT6s levels in MG among the different conditions, no significant differences were found. It could be anticipated that the levels would be significantly higher at night of the nonstressful task, based on literature data that relaxation states (e.g., meditation practice) are associated with increased secretion of MLT (Harinath et al., 2004; Liou et al., 2010; Massion, Teas, Hebert, Wertheimer, & Kabat‐Zinn, 1995; Tooley, Armstrong, Norman, & Sali, 2000), while more aroused states are associated with lower MLT secretion (Arnetz & Berg, 1996; Monteleone, Fuschino, Nolfe, & Maj, 1992). On the other hand, it could be assumed that the levels would be higher in the night of mediumistic experience, considering the speculation that PG would play an important role for human transcendent experiences (Lucchetti et al., 2013; Luke, 2011; Strassman, 2001). However, neither of these two propositions has been confirmed. These results lead us to state that the conjectured relationship of PG with spiritual experiences, if any, should be done on other (yet unexplored) bases other than through the production of MLT (e.g., secretion of hallucinogenic tryptamines, and magneto receptors) (Baconnier et al., 2002; Barker, 2018; Bastos, Bastos, Dos Santos, et al., 2018).

### 3.6 Stress, spirit possession and the influence of cultural factors

Dissociation can be defined, in short, as a lack of integration among two or more different “systems of ideas and functions that constitute personality” (Janet, 1889) and most scholars view it predominantly as a consequence of previous traumatic experiences, consisting in a psychological defense mechanism (Seligman & Kirmayer, 2008). However, some authors deem this as a too reductionist notion and demand the adoption of a more comprehensive approach, to include cultural aspects (Maraldi, Ribeiro, & Krippner, 2019; Seligman & Kirmayer, 2008; Somer, 2006).

These scholars advocate that a tendency to dissociate appears to be present in some individuals since childhood, preceding traumatic experiences (Maraldi et al., 2019; Seligman & Kirmayer, 2008). Gradually, these subjects learn that the lack of cortical integration of cognitive processes may serve as a coping strategy, and many put it into action during times of acute stress. Furthermore, certain cultural contexts (e.g., Spiritism and Balinese religions) can value, reinforce and shape these dissociative occurrences, often contributing to the integration of these events in their personalities in a healthy way (Maraldi et al., 2019; Seligman, 2005; Seligman & Kirmayer, 2008). However, in some cases, dissociative experiences occur in an uncontrolled manner, in socially unappropriated places and times. This is an afflictive condition, because in such cases even trivial acute stress present in daily life triggers the dissociative experience. This picture characterizes pathological dissociation, in which subjects have cognitive and social malfunction, experience chronic stress, and have their physical health impaired in the long term (Seligman & Kirmayer, 2008). Hence, we believe that the mediums in our sample have dissociative experiences of a normative type, not only because they report having good control over such experiences, but also because they showed a normal pattern of emotion regulation in response to an acute artificial stress (TSST).

Finally, during the nonstressful control task (performed in equivalent form in both groups), significant changes indicative of mental and physical relaxation, as assessed by anxiety state and heart rate, were observed in the MG, but not in the CG. This may point to the presence of mediums' greater absorption by the story, which was being read, allowing this to have a greater influence on their mental and physical states. Some researchers argue that mediums would be individuals with a greater tendency toward somatization and embodiment (Greenfield, 1994; Seligman, 2005; Seligman & Halloy, 2017), and our results seem to corroborate such theories. Oxytocin could be related to these characteristics because it is a neuropeptide synthesized by hypothalamic neurons with a relevant role for social behavior in humans (Bartz, Zaki, Bolger, & Ochsner, 2011). Evidence indicates that intranasal administration of exogenous oxytocin in humans attenuates anxiety and decreases stress reactivity (Heinrichs, Baumgartner, Kirschbaum, & Ehlert, 2003), as well as leads to an increase in self‐reported spirituality scores (Van Cappellen, Way, Isgett, & Fredrickson, 2016). In addition, higher levels of endogenous salivary oxytocin are positively associated with higher spirituality scores (Holbrook, Hahn‐Holbrook, & Holt‐Lunstad, 2015). Future studies should investigate the possible relationship of oxytocin with mediumistic experiences.

### 3.7 Limitations of the study and future directions

The present study has some limitations. First, our studies included small sample sizes. Nevertheless, sample size calculation was performed and detected enough statistical power using this sample size. Second, there is a lack of information regarding participants' history of trauma. Events such as child physical or sexual abuse are thought to have an etiological role for dissociation so, although the present study has focused culture‐bound dissociation, this should also have been assessed. Third, we had technical difficulty in determining pineal cyst volumes (ideally, the volume of the pineal parenchyma should have been calculated by subtracting the cyst volume from the total pineal volume), so we resigned to include this measure. In addition, calculating the volume of pineal gland and melatonin level is obviously not enough to clarify the putative role of the pineal gland in spirit possession, but we believe that the present findings may help to direct future research. Further clarification of the neurobiological mechanism of spirit possession may require the investigation of neural circuits and neurotransmitter systems. In view of the altered state and scope of consciousness during spirit possession, we believe that forthcoming studies should explore other key brain regions closely related to the state of consciousness, such as the ascending activation system of midbrain and prefrontal cortex. Finally, the physiological and neurostructural correlates of spirit possession may differ between young individuals with recent onset of anomalous sensory experiences (which they are unable to control, bringing affliction) and experienced, socially integrated mediums. So, prospective studies comparing these parameters at different time points throughout the natural history of their conditions would be valuable.

## 4 CONCLUSION

In sum, the findings of the present study indicate that individuals with psychotic‐like symptoms linked to the cultural context (in this case, Spiritist mediums) have normal secretory and structural characteristics of PG, quite different from the picture usually found in individuals with psychotic disorder. Yet, an increase in MLT production does not appear to be a distinguishing feature of mediumistic experience. Additionally, mediums presented a fast recovery stress response pattern, indicating good emotional regulation, unlike patients with pathological dissociation. Hence, the main implication of this study is to reinforce the notion of mediumship as a nonpathological dissociative phenomenon. This underscores the importance of refining the differential diagnosis between nonpathological forms of spiritual possession versus psychotic and dissociative disorders, as evidence increasingly demonstrates that culturally well‐integrated mediums are often mentally and physically healthy. Thus, the correct differentiation between these conditions could avoid stigmatization and unnecessary treatments.

## CONFLICT OF INTEREST

The authors declare no conflict of interest.

## AUTHOR CONTRIBUTIONS

MAVBJ, PRHO, GL, RTP, RBP, and DIJ designed the study. MAVBJ, LEFP, EOS, RBP, RTP, DB, and JGOO executed the data collection. GL and MAVBJ provided the statistical analyzes. MAVBJ wrote the first draft of the manuscript and the other authors revised it. All authors provided critical feedback, contributed to the interpretation of the results, helped to shape, and approved the final manuscript.

## Supporting information

Supplementary Material

## Data Availability

The data that support the findings of this study are available from the corresponding author upon reasonable request.
