# Supplementary material for: “Seat of the soul”? The structure and function of the pineal gland in women with alleged spirit possession—Results of two experimental studies
Source: Brain Behav. 2020 Jun 7;10(7):e01693. doi: 10.1002/brb3.1693 (PMC7375051; doi:10.1002/brb3.1693)
Supplement: Supplementary file 1 — Supplementary Material [file BRB3-10-e01693-s001.docx]

‘Seat of the souls’? The structure and function of the pineal gland in women with alleged spirit possession – results of two experimental studies

**Supplementary Material**

**SUPPLEMENTARY TABLE 1**

Absolute and relative frequency of participants who endorsed regularly presenting each anomalous experience (adapted from Menezes et al., 2013) in the medium (MG) and control (CG) groups

|  | **Group** | |  |
| --- | --- | --- | --- |
|  | Mediums  (n=16) | Controls  (n=16) | **Statistic** **(p^+^)** |
| Apparitional experiences | 7 (43.8%) | 1 (6.2%) | **0.037*** |
| Spiritual hearing | 5 (31.2%) | 1 (6.2%) | 0.086 |
| Spiritual perception | 15 (93.8%) | 8 (50%) | **0.015*** |
| Abnormal dreams | 10 (62.5%) | 2 (12.5%) | **0.009*** |
| Out-of-body experiences | 5 (31.2%) | 4 (25%) | 0.500 |
| Foretelling | 6 (37.5%) | 5 (31.2%) | >0.999 |
| Unexplained loss of energy | 7 (43.8%) | 5 (31.2%) | 0.716 |
| Possession | 7 (43.8%) | 0 (0%) | **0.007*** |
| Intuition | 13 (81.2%) | 7 (43.8%) | 0.066 |
| Spiritual perception of odors | 4 (25%) | 4 (25%) | >0.999 |
| Physical manifestations of spiritual cause | 0 (0%) | 1 (6.2%) | >0.999 |
| Psychography | 6 (37.5%) | 0 (0%) | **0.018*** |
| Telepathy | 6 (37.5%) | 3 (18.8%) | 0.433 |
| Spiritual healing | 5 (31.2%) | 4 (25%) | >0.999 |

^+^ Fisher`s Exact test, two-tailed/ ***** p<0.05

**SUPPLEMENTARY TABLE 2**

Between**-**group comparisons regarding sociodemographic factors

|  | | Group | | **Statistic** (Qui Square test) |
| --- | --- | --- | --- | --- |
|  |  | **Mediums**  (n=16) | **Controls**  (n=16) |  |
| **Ethnicity** | White | 10 (62.5%) | 9 (56.2%) | p=0.719 |
|  | Nonwhite | 6 (37.5%) | 7 (43.8%) |  |
| **Education** | University | 13 (81.2%) | 12 (75%) | p=0.669 |
|  | Less than University | 3 (18.8%) | 4 (25%) |  |
| **Marital status** | Married | 7 (43.8%) | 15 (93.8%) | p=0.002* |
|  | Unmarried | 9 (56.2%) | 1 (6.2%) |  |
| **Spiritualist Religion** | Yes | 16 (100%) | 0 (0%) | p<0.001 |
|  | No | 0 (0%) | 16 (100%) |  |

**SUPPLEMENTARY TABLE 3**

Correlations between marital status and religion with the scores on the psychometric scales evaluated in the total sample (n = 32) - correlation coefficient (p value ^d^)

|  | **Marital status** | **Religion** |
| --- | --- | --- |
|  | Correlation coefficient (p) ^d^ | Correlation coefficient (p) ^d^ |
| **Anomalous experiences** | 0.14  (0.440) | **0.64**  **(<0.001)*** |
| **SRQ ^a^** | 0.21  (0.237) | **0.36**  **(0.042)*** |
| **DES ^b^** | 0.28  (0.114) | 0.17  (0.334) |
| **DES – absorption** | 0.09  (0.621) | 0.15  (0.410) |
| **DES – depersonalization** | 0.31  (0.083) | **0.36**  **(0.044)*** |
| **DES – amnesia** | 0.18  (0.320) | 0.05  (0.787) |
| **PSQI ^c^** | 0.16  (0.368) | 0.17  (0.340) |

^a^ Self-Report Psychiatric Screening Questionnaire/ ^b^ Dissociative Experiences

Scale/ ^c^ Pittsburgh Sleep Quality Index/ ^d^ Spearman`s test/ ***** p<0.05

**SUPPLEMENTARY TABLE 4**

Anthropometric aspects in medium group (MG) and control group (CG)

|  | **Group** | |  |  |
| --- | --- | --- | --- | --- |
|  | Mediums (n=16) | Controls (n=16) | **p^c^** | ***Effect size (d) ^d^*** |
|  | Mean (SD)^b^ | Mean (SD) |  |  |
| **BMI^a^ (kg/m^2^)** | 25.1 (2.7) | 26.4 (3.2) | 0.231 | 0.439 |
| **Cephalic perimeter (cm)** | 54.6 (1.5) | 54.3 (0.9) | 0.517 | 0.242 |

^a^ Body mass index/ ^b^ Standard deviation/ ^c^ Student`s t test/ ^d^ Cohen`s d

**SUPPLEMENTARY TABLE 5**

Intergroup comparison regarding participants' handedness and frequency of alcohol consumption

|  | | **Group** | | **Statistic** (Qui Square test) |
| --- | --- | --- | --- | --- |
|  |  | Mediums  (n=16) | Controls  (n=16) |  |
| **Handedness** | Right-handed | 14 (87.5%) | 16 (100%) | p=0.144 |
|  | Left-handed | 2 (12.5%) | 0 (0%) |  |
| **Alcohol consumption** | Up to once/ month | 13 (81.2%) | 6 (37.5%) | p=0.012* |
|  | ≥ 2 times/ month | 3 (18.8%) | 10 (62.5%) |  |

**SUPPLEMENTARY FIGURE 1**

Image illustrating magnetic resonance (MR) measurement of the mid-sagittal intracranial area of one of the research participants

**SUPPLEMENTARY FIGURE 2**

No diferences in mid-sagittal intracranial area between the medium group (MG) and the control group (CG)

**SUPPLEMENTARY FIGURE 3**

Images exemplifying magnetic resonance (MR) measurement of research participant's pituitary gland (PG) volume

**SUPPLEMENTARY FIGURE 4**

No diferences in pituitary gland volume between the medium group (MG) and the control group (CG)

**SUPPLEMENTARY TABLE 6**

Inter-examiner correlations of measurements of structural neuroimaging parameters

|  | **R** | **p**^+^ |
| --- | --- | --- |
| **Mid-sagittal intracranial area** | 0.88 | 0.001***** |
| **Pineal gland volume** | 0.91 | <0.001***** |
| **Pituitary gland volume** | 0.90 | <0.001***** |

^+^ Spearman`s test/ ***** p<0.05

**SUPPLEMENTARY TABLE 7**

Correlations between frequency of alcohol consumption and age with variables assessed by structural brain magnetic resonance imaging (MRI), in the medium group (MG) and the control group (CG)

|  | | **Alcohol consumption** | **Age** |
| --- | --- | --- | --- |
|  |  | Correlation coefficient (p) ^a^ | Correlation coefficient (p) ^a^ |
| **Mid-sagittal intracranial area** | MG | -0.20  (0.447) | -0.46  (0.074) |
|  | CG | 0.36  (0.170) | -0.09  (0.734) |
| **Pineal gland volume** | MG | 0.28  (0.287) | -0.22  (0.421) |
|  | CG | 0.07  (0.788) | 0.17  (0.516) |
| **Pituitary gland volume** | MG | 0.09  (0.739) | **-0.64**  **(0.007)*** |
|  | CG | **0.51**  **(0.042)*** | -0.20  (0.450) |

^a^ Spearman`s test/ ***** p<0.05

**SUPPLEMENTARY FIGURE 5**

Psychophysiological parameters, on the night of sample collection (non-stressful task-control), in the medium (MG) and control (CG) groups. Absence of statistically significant differences between groups. Significant reduction in heart rate (within-group) noted only in MG. Dots represent the means and vertical bars the standard errors.

**SUPPLEMENTARY TABLE 8**

Correlations between 6-sulfatoxymelatonin and other relevant variables in the medium group (MG) and control group (CG) - correlation coefficient (p value^a^)

|  | | **6-Sulfatoxy-melatonin** |
| --- | --- | --- |
| **Alcohol consumption** | MG | 0.48  (0.057) |
|  | CG | 0.28  (0.345) |
| **Age** | MG | 0.25  (0.347) |
|  | CG | 0.02  (0.943) |
| **Pineal gland volume** | MG | **0.61**  **(0.012)*** |
|  | CG | 0.04  (0.902) |
| ***PSQI*^b^** | MG | 0.09  (0.727) |
|  | CG | -0.41  (0.166) |

^a^ Spearman`s test/ ^b^ *Pittsburgh Sleep Quality Index/* *****p<0.05
